# Combined bead polymerization and *Cinchona* organocatalyst immobilization by thiol–ene addition

**DOI:** 10.3762/bjoc.8.125

**Published:** 2012-07-20

**Authors:** Kim A Fredriksen, Tor E Kristensen, Tore Hansen

**Affiliations:** 1 Department of Chemistry, University of Oslo, P.O. Box 1033 Blindern, NO-0315 Oslo, Norway; 2 Norwegian Defence Research Establishment (FFI), P.O. Box 25, NO-2027 Kjeller, Norway

**Keywords:** asymmetric catalysis, *Cinchona* derivatives, organocatalysis, polymerization, thiol–ene reaction

## Abstract

In this work, we report an unusually concise immobilization of *Cinchona* organocatalysts using thiol–ene chemistry, in which catalyst immobilization and bead polymerization is combined in a single step. A solution of azo initiator, polyfunctional thiol, polyfunctional alkene and an unmodified *Cinchona*-derived organocatalyst in a solvent is suspended in water and copolymerized on heating by thiol–ene additions. The resultant spherical and gel-type polymer beads have been evaluated as organocatalysts in catalytic asymmetric transformations.

## Introduction

Polymer-supported chiral organocatalysts have emerged as a rapidly expanding field of research in recent years [1], in part due to the traditionally emphasized advantages of polymeric immobilization (facilitated separation and recovery procedures, recycling etc.), but perhaps even more due to the enhanced activity and selectivity sometimes exhibited by such organocatalysts, especially under aqueous conditions [2]. Recently, the use of polymer-supported organocatalysts in continuous-flow systems has also surfaced in the literature, and their development is quickly gaining momentum [3–5]. Regrettably, cost issues linger over the field as a whole, due to the lengthy and laborious syntheses involved in the preparation of these polymer-supported entities, hampering the more widespread utilization of polymer-supported reagents or catalysts as part of conventional chemical synthesis.

Consequently, we have been engaged in the development of scalable and expedient syntheses of polymer-supported organocatalysts for some time now [6–7]. In our bottom-up approach for the preparation of polymer-supported organocatalysts, the catalyst immobilization and the preparation of the polymer scaffold are closely connected, to facilitate the synthesis of larger quantities of supported catalyst [6–7]. Acrylic derivatives of established organocatalysts are prepared on a gram scale by using nonchromatographic procedures and copolymerized with suitable comonomers to give cross-linked and microporous beads. Such polymer beads have provided good to excellent results as organocatalysts in various asymmetric transformations [6–7].

*Cinchona* derivatives are used in several types of organocatalysts, and they are all equipped with a pendant vinylic functionality susceptible to activation by chemical transformations based on radical intermediates [1]. As a result, polymeric immobilization of *Cinchona* derivatives by using thiol–ene addition has a substantial history, founded on procedures developed already in the early 1970s [1]. *Cinchona* derivatives are either copolymerized with certain comonomers, such as acrylonitrile, directly in a bottom-up fashion to give linear copolymers [1,8], or anchored to prefabricated cross-linked and thiol-funtionalized resins in a traditional post-modification approach [1,9]. However, for the preparation of the preferred beaded and cross-linked polymer resins, so easily handled and separated from reaction mixtures by filtration, this necessitates several steps, as the cross-linked resin must be prepared first by copolymerization, then equipped with thiol functionalities, and finally joined with the *Cinchona* derivative through thiol–ene coupling.

The thiol–ene addition was described by Theodor Posner already in 1905 [10], and it has been in more or less continuous use since then. The thiol–ene addition can readily be adapted for polymerization, by using polyfunctional alkenes in combination with polyfunctional thiols [11–13]. We envisaged that polymerization into a cross-linked and beaded resin could be combined with immobilization of a *Cinchona* derivative in a single step under suitable conditions. Such a procedure would enable us to prepare polymer-supported *Cinchona* organocatalysts directly in a single step and on a large scale, using unmodified *Cinchona* organocatalyst precursors.

## Results and Discussion

### Building blocks for the preparation of cross-linked thiol–ene resins

**Figure 1:**
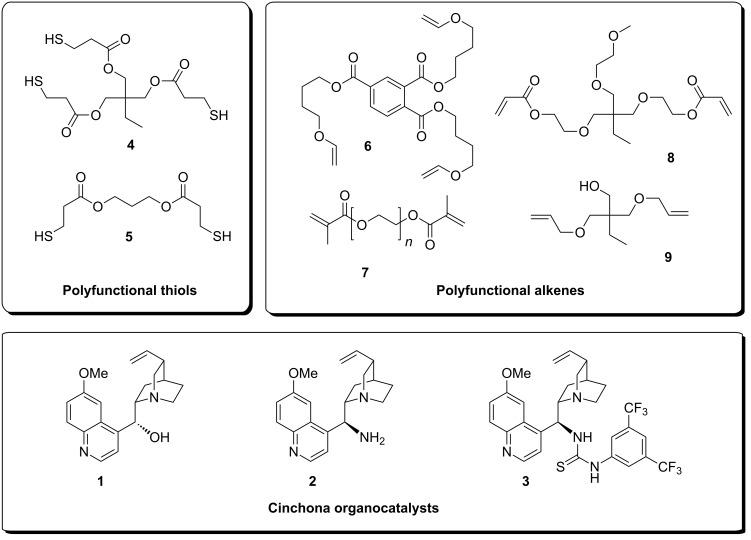
Thiol, alkene and organocatalyst building blocks for combined bead polymerization and *Cinchona* organocatalyst immobilization.

Research oriented towards thiol–ene chemistry has experienced near explosive growth in the past few years, perhaps due to its efficiency and functional tolerance, but possibly even more due to its recent conceptualization as a “click” reaction [13]. In order to prepare different polymer beads with varying degrees of swelling characteristics, we assembled a small collection of useful thiol and alkene building blocks (Figure 1).

Trithiol **4** is a readily available commercial product, whereas dithiol **5** was easily obtained from esterification of 3-mercaptopropionic acid and propane-1,3-diol [14]. Trivinyl ether **6**, polyethylene glycol (PEG) dimethacrylate **7**, diacrylate **8** and diallyl ether **9** are all commercially available compounds. For thiol–ene additions via the radical pathway, the order of reactivity of unsaturated compounds **6**–**9** towards the thiols is generally so: vinyl ether **6** > allyl ether **9** > acrylate **8** > methacrylate **7** [11]. Thiol–ene additions according to the anionic (Michael-type) mechanism have not been investigated in this work. As a minimum for obtaining cross-linked polymeric networks, either trithiol **4** has to be copolymerized with dialkenes **7**–**9**, or dithiol **5** has to be copolymerized with trivinyl ether **6**. These two main approaches can then be modified or finely tuned to match suitable swelling characteristics by incorporation of smaller amounts of any of the other constituents **4**–**9**, thereby adjusting the degree of cross-linking.

As for the *Cinchona* organocatalysts, we wanted to incorporate either unmodified quinine (**1**), the primary amine organocatalyst **2**, or thiourea organocatalyst **3** into the thiol–ene network (Figure 1). While quinine is available directly, primary amine organocatalyst **2** was prepared from quinine, via the azide, in a two-step sequence by using the Bose–Mitsunobu reaction followed by Staudinger reduction, as described by others [15]. Thiourea *Cinchona* organocatalyst **3** was easily obtained from catalyst **2** by reaction with the appropriate aromatic isothiocyanate [15].

### Single step thiol–ene polymerization and *Cinchona* organocatalyst immobilization

**Scheme 1:**
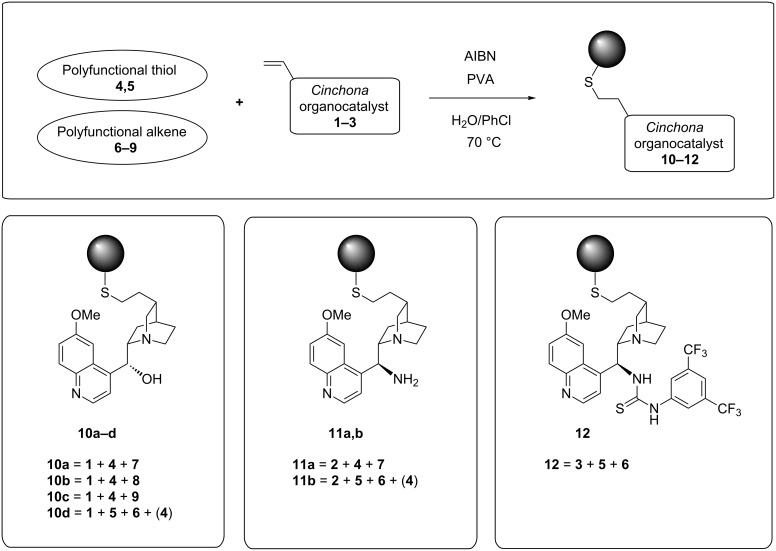
Combined bead polymerization and *Cinchona* organocatalyst immobilization by thiol–ene addition.

With the assortment of building blocks depicted in Figure 1 available, we could now obtain immobilized versions (**10**–**12**) of unmodified *Cinchona* catalysts **1**–**3** directly by oil-in-water type thiol–ene suspension copolymerization. A solution of polyfunctional thiol, polyfunctional alkene and *Cinchona* organocatalyst in a water immiscible solvent, such as chlorobenzene or toluene, containing a small amount of azo radical initiator (AIBN), was suspended in dilute aqueous polyvinyl alcohol (PVA) and heated under vigorous agitation. The suspended and stabilized droplets then converted to spherical and gel-type polymer beads. An overview of the immobilized *Cinchona* organocatalysts, and their constituents, is provided in Scheme 1.

After polymerization, the polymer beads were filtered and purified by Soxhlet extraction, and their organocatalyst loadings were determined on the basis of CHN analysis, as only the organocatalytic moiety contains nitrogen. Generally, the yield of polymer beads, calculated on the basis of recovered material versus the combined mass of starting materials, varied between ca. 60–80%. Only an azo initiator, such as AIBN, could be utilized, because peroxide initiators, such as dibenzoyl peroxide, oxidise thiols.

The thiol-ene polymer beads **10**–**12** had a distinctively soft and gel-like appearance, but were easily handled like conventional microporous beads. They had favourable swelling characteristics in several organic solvents, particularly in THF and CH_2_Cl_2_, despite their significant degree of cross-linking. As such, they have much in common with the CLEAR (cross-linked ethoxylate acrylate resin) resins, a type of polymer support with all the characteristics of a microporous polymer that also has an unusually high degree of cross-linking [16]. Unlike vinyl ether **6** and allyl ether **9**, acrylic building blocks such as **7** and **8** may undergo some degree of acrylic homopolymerization during network formation, although the thiol–ene addition is usually more rapid than the polymerization.

The ratio of thiol and alkene functionalities was adjusted to be close to unity, and the thiol–ene reaction usually has a high degree of conversion; however, the presence of free thiol groups is probably unavoidable. We were, nevertheless, curious to investigate how these supported organocatalysts would function in asymmetric transformations when compared to the unsupported catalysts.

### Asymmetric organocatalytic transformations using immobilized *Cinchona* organocatalysts

With supported *Cinchona* organocatalysts **10**–**12** available, numerous organocatalytic transformations were potentially available for benchmarking. As a rough indication of activity, we started out by investigating quinine (**1**) and supported catalyst **10a**–**d** in the Michael addition of 3-methoxythiophenol and cyclohex-2-enone (Table 1) [17]. Although the performance of quinine in this reaction is poor with regards to selectivity (providing only 23% ee), and probably not very useful for benchmarking, the supported catalysts **10a**–**d** were obviously catalytically active, albeit modestly selective compared to the free catalyst, giving quantitative yields and a selectivity of 11–14% ee.

**Table 1:** Polymer-supported quinines in asymmetric Michael addition.

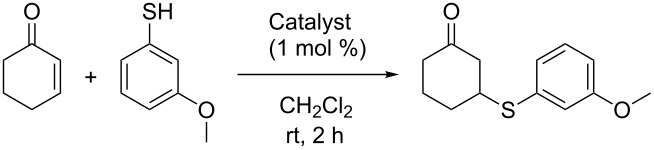		

Catalyst	Yield [%]^a^	ee [%]^b^

**1**	>95	23
**10a**	>95	14
**10b**	>95	11
**10c**	92	14
**10d**	>95	12

^a^Isolated yield. ^b^Determined by HPLC analysis.

Of greater interest was the performance of the primary amine organocatalysts **11**. Polymer-supported catalysts **11a**,**b** were tried out in the asymmetric preparation of the anticoagulant warfarin from benzylideneacetone and 4-hydroxycoumarin, a transformation that we have investigated in our group as part of developmental work in primary amine organocatalysis on a previous occasion (Table 2) [18]. Compared to the free catalyst **2**, the yields obtained by using supported catalysts **11a**,**b** are inferior, but this is most probably due to the lack of solubility of the hydroxycoumarin, a very insoluble compound, in the reaction medium (CH_2_Cl_2_) and probably not so much a lack of inherent activity. Interestingly, catalyst **11b**, made by using dithiol **5** and trivinyl ether **6** (in addition to a few mol % of trithiol **4** to increase cross-linking slightly, giving beads of better quality), exhibited markedly improved selectivity compared to catalyst **11a**, even matching that of the free catalyst **2**. This may be connected to the fact that the *Cinchona* moiety becomes bound to the polymer network through the thiol, and use of a difunctional thiol then improves the mobility of this catalytic unit as it is now positioned at the end of a linker of greater length. This effect does not seem to have played out for catalyst **10d** though. Catalysts **10a**–**d** all seemed to behave much the same. Catalyst **11b** could be recycled, but both yield and selectivity quickly eroded (Table 2).

**Table 2:** Polymer-supported primary amine *Cinchona* organocatalysts in asymmetric preparation of warfarin.

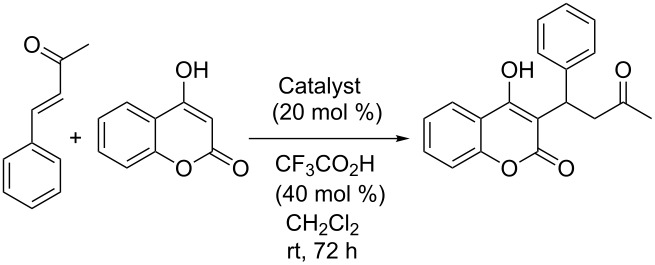		

Catalyst	Yield [%]^a^	ee [%]^b^

**2**	75	92
**11a**	15	77
**11b**^c^	25	94
**11b**^d^	15	84
**11b**^e^	—	—

^a^Isolated yield. ^b^Determined by HPLC analysis. ^c^First cycle. ^d^Second cycle. ^e^Third cycle.

Having confidence in the overall validity of our approach to polymer-supported *Cinchona* organocatalysts, we tested the supported thiourea catalyst **12** in the Michael addition of thiophenol and cyclohex-2-enone [17], a transformation known to be very efficiently catalysed by the free thiourea catalyst **3** (Table 3) [19–20]. The immobilized catalyst **12** proved highly active (the uncatalysed reaction is very slow), rapidly giving quantitative yield, but somewhat reduced selectivity of the addition product compared to the free thiourea catalyst **3**. To investigate the extent to which free thiol groups could influence the reaction by catalyzing a racemic pathway, we also tested polymer beads without any *Cinchona* moiety present in this transformation. Undeniably, polymer beads prepared without any *Cinchona* organocatalyst present did also catalyze the reaction. However, we found that several other polymer resins, such as unmodified Merrifield resin (chloromethylated and cross-linked polystyrene), influence the reaction in the same manner. Consequently, we do not believe this to be an intrinsic property of our thiol–ene polymer beads, connected to the effect of free thiol groups. In addition, capping free thiol groups on beforehand by treatment with excess methyl acrylate did not affect the performance in this transformation.

**Table 3:** Polymer-supported thiourea *Cinchona* organocatalyst in the asymmetric Michael addition.

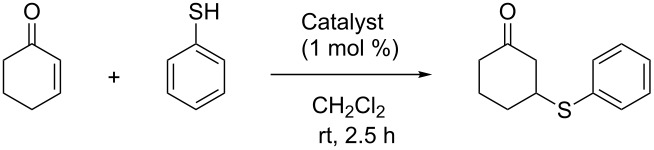		

Catalyst	Yield [%]^a^	ee [%]^b^

**3**	>95	83
**12**	>95	69

^a^Isolated yield. ^b^Determined by HPLC analysis.

Catalyst **12** was also tested in the Michael addition of methyl malonate to *trans*-β-nitrostyrene (Table 4) [21]. The catalyst gave quantitative yield and excellent enantioselectivity after 3–4 days reaction time. However, the catalyst exhibited poor recycling properties as yields fell sharply after the second reaction cycle; but selectivity remained largely untouched. At this point, we suspected that loss of the catalytic entities from the polymer resin may explain this, and indeed, CHN analysis of polymer resins after recycling verified that a loss of nitrogen content, meaning leaching of the active species, had occurred.

**Table 4:** Polymer-supported thiourea *Cinchona* organocatalyst in the asymmetric Michael addition.

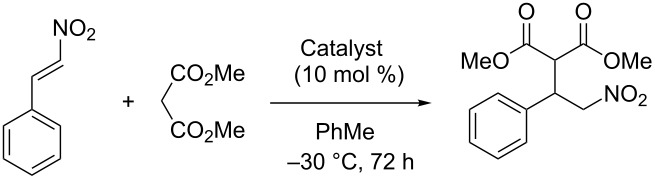		

Catalyst	Conversion [%]^a^	ee [%]^b^

**12**^c^	>95	92
**12**^d^	>95	92
**12**^e^	34	92
**12**^f^	trace	—

^a^Determined by ^1^H NMR analysis of crude reaction mixture. ^b^Determined by HPLC analysis. ^c^First cycle, 3 d reaction time. ^d^Second cycle, 4 d reaction time. ^e^Third cycle, 4 d reaction time. ^f^Fourth cycle, 4 d reaction time.

## Conclusion

We have developed an unusually concise immobilization of *Cinchona* organocatalysts by thiol–ene suspension copolymerization of polyfunctional thiols and alkenes together with unmodified *Cinchona* organocatalyst precursors. As such, bead polymerization and catalyst immobilization is combined in a single step. Vinyl ethers, allyl ethers, acrylates and methacrylates can all be effectively incorporated as part of such thiol–ene networks. The supported organocatalysts have been tried out successfully in several asymmetric transformations, but catalyst recycling so far is relatively poor. Hopefully, this expedient method for immobilization of *Cinchona* derivatives can be further developed in the future to improve activity and selectivity, and also be widened to include other useful *Cinchona*-derived species.

## Experimental

**General**: ^1^H NMR and ^13^C NMR spectra were recorded on a Bruker AV 600 (600/150 MHz), Bruker Advance DRX 500 (500/125 MHz), Bruker DPX 300 (300/75 MHz) or Bruker DPX 200 (200/50 MHz) spectrometer. Dry THF was obtained from a solvent purification system (MB SPS-800 from MBraun). All other reagents and solvents were used as received. CHN analyses were carried out in the School of Chemistry, at the University of Birmingham, UK. For flash chromatography, silica gel from SdS (60 A, 40–63 μm, 550 m^2^/g, pH 7) and Merck (silica gel 60, 0.40–0.63 mm, 480–540 m^2^/g, pH 6.5–7.5) were used, either manually or with an automated system (Isco Inc. CombiFlash Companion with PeakTrak software), with EtOAc/hexanes of technical quality. Enantiomeric excess was determined by HPLC analysis using analytical columns (Chiralpak AS-H or AD-H from Daicel Chemical Industries).

The *Cinchona* organocatalysts **2** and **3** were prepared from quinine (**1**) as described in the literature [15]. Dithiol **5** was prepared as described in the literature [14], and this compound was kept refrigerated and protected from light in order to avoid deterioration on storage.

**Preparation of polymer-supported quinines 10a**–**d**: Quinine (**1**, 2.10 mmol for **10a** or 2.00 mmol for **10b**/**10c** or 1.50 mmol for **10d**), trithiol **4** (6.20 mmol for **10a**/**10c** or 6.00 mmol for **10b** or 0.18 mmol for **10d**), dithiol **5** (4.00 mmol for **10d**), dimethacrylate **7** (8.90 mmol for **10a**), diacrylate **8** (8.10 mmol for **10b**), diallyl ether **9** (8.10 mmol for **10c**), trivinyl ether **6** (2.43 mmol for **10d**) and AIBN (5 wt % relative to monomers) were dissolved in a monomer diluent (13 mL PhCl for **10a** or 15 mL PhCl for **10b**/**10c** or 5 mL PhCl for **10d**). Aqueous PVA (100 mL for **10a** or 70 mL for **10b**/**10c** or 39 mL for **10d**, 0.5% Mowiol 40-88) was added under stirring to give an oil-in-water type emulsion. The system was flushed with argon for 5 min. The suspension was heated to 70 °C and kept at this temperature for 3 h under stirring, allowed to cool to room temperature, and poured into a beaker containing MeOH (250 mL). The suspension was stirred for 15 min, and the polymer beads were allowed to settle by gravity for 10 min. The supernatant was removed by decantation and the process was repeated until the supernatant was transparent (1–2 repetitions). CH_2_Cl_2_ (50 mL) was added, the suspension was filtered by vacuum, and the polymer beads were washed with water (1000 mL), THF–H_2_O (200 mL, 1:1), MeOH (200 mL) and CH_2_Cl_2_ (50 mL). The polymer beads were then transferred to a cellulose paper thimble and purified by Soxhlet extraction with CH_2_Cl_2_ (70 mL) for 12 h, and then the purified beads were left to dry at room temperature for 24 h (67% yield for **10a**, 67% yield for **10b**, 63% yield for **10c**, 64% yield for **10d**). Catalyst loadings were determined by CHN analysis.

**Preparation of polymer-supported primary amine *Cinchona* organocatalysts 11a**,**b**: *Cinchona* derivative **2** (2.00 mmol for **11a** or 1.20 mmol for **11b**), trithiol **4** (5.00 mmol for **11a** or 0.16 mmol for **11b**), dithiol **5** (4.20 mmol for **11b**), dimethacrylate **7** (6.24 mmol for **11a**), trivinyl ether **6** (2.51 mmol for **11b**) and AIBN (5 wt % relative to monomers) were dissolved in a monomer diluent (12 mL PhCl for **11a** or 6 mL PhMe for **11b**). Aqueous PVA (70 mL for **11a** or 40 mL for **11b**, 0.5% Mowiol 40-88) and potassium iodide (to inhibit polymerization in the aqueous phase, 23 mg for **11b**) was added under stirring to give an oil-in-water type emulsion. The system was flushed with argon for 5 min. The suspension was heated to 70 °C and kept at this temperature for 3 h under stirring, allowed to cool to room temperature, and then poured into a beaker containing MeOH (250 mL). The suspension was stirred for 15 min, and the polymer beads were allowed to settle by gravity for 10 min. The supernatant was removed by decantation, and the process was repeated until the supernatant was transparent (1–2 repetitions). CH_2_Cl_2_ (50 mL) was added, the suspension was filtered by vacuum, and the polymer beads washed with water (1000 mL), THF–H_2_O (200 mL, 1:1), MeOH (200 mL) and CH_2_Cl_2_ (50 mL). The polymer beads were then transferred to a cellulose paper thimble and purified by Soxhlet extraction with CH_2_Cl_2_ (70 mL) for 12 h, and then the purified beads were left to dry at room temperature for 24 h (75% yield for **11a**, 85% yield for **11b**). Catalyst loadings were determined by CHN analysis.

**Preparation of polymer-supported thiourea *Cinchona* organocatalyst 12**: *Cinchona* derivative **3** (0.538 mmol), trithiol **4** (0.17 mmol), dithiol **5** (3.48 mmol), trivinyl ether **6** (2.32 mmol) and AIBN (15 wt % relative to monomers) were dissolved in PhMe (7 mL). Aqueous PVA (50 mL, 0.5% Mowiol 40-88) and potassium iodide (to inhibit polymerization in the aqueous phase, 20 mg) was added under stirring to give an oil-in-water type emulsion. The system was flushed with argon for 5 min. The suspension was heated to 70 °C and kept at this temperature for 1 h under stirring, allowed to cool to room temperature, and poured into a beaker containing MeOH (250 mL). The suspension was stirred for 15 min, and the polymer beads were allowed to settle by gravity for 10 min. The supernatant was removed by decantation, and the process was repeated until the supernatant was transparent (1–2 repetitions). CH_2_Cl_2_ (50 mL) was added, the suspension was filtered by vacuum, and the polymer beads washed with water (1000 mL), THF–H_2_O (200 mL, 1:1), MeOH (200 mL) and CH_2_Cl_2_ (50 mL). The polymer beads were then transferred to a cellulose paper thimble and purified by Soxhlet extraction with CH_2_Cl_2_ (70 mL) for 12 h, and the purified beads were left to dry at room temperature for 24 h (62% yield). Catalyst loadings were determined by CHN analysis.

**General procedure for asymmetric Michael addition of 3-methoxythiophenol to cyclohex-2-enone**: 3-Methoxythiophenol (0.30 mL, 3.10 mmol), and catalyst (1 mol %) were dissolved in CH_2_Cl_2_ (6 mL). Cyclohex-2-enone (0.50 mL, 4.03 mmol) was then added in one portion, and the resulting mixture was stirred at room temperature for 2 h. The crude reaction mixture was filtered, and the polymer beads were washed with CH_2_Cl_2_ (50 mL). The combined organic phase was evaporated in vacuo, and the crude product was purified by flash chromatography on silica gel (10% EtOAc in hexanes) to give the product as a colorless oil. This is a known compound [17]. Enantiomeric excess was determined by HPLC analysis (Chiralpak AS-H, 50% iPrOH in isohexane, 0.3 mL/min): *t*_R_ = 25.2 min and 38.2 min.

**General procedure for asymmetric Michael addition of 4-hydroxycoumarin to benzylideneacetone**: To a vial containing 4-hydroxycumarin (0.35 g, 2.17 mmol), benzylideneacetone (0.56 g, 3.82 mmol) and catalyst (20 mol %), was added CH_2_Cl_2_ (18 mL) and CF_3_CO_2_H (60 μL, 40 mol %). The reaction mixture was stirred at room temperature for 72 h, diluted with CH_2_Cl_2_ and filtered. The polymer beads were washed with CH_2_Cl_2_ (70 mL). The combined organic phase was evaporated in vacuo, and the crude product was purified by flash chromatography on silica gel (10% EtOAc in hexanes and then 25% EtOAc in hexanes) to give the product as a colorless solid. This is a known compound [18]. Enantiomeric excess was determined by HPLC analysis (Chiralpak AD-H, 20% iPrOH in isohexane, 1.0 mL/min): *t*_R_ = 7.3 min and 14.7 min.

**General procedure for asymmetric Michael addition of thiophenol to cyclohex-2-enone**: Thiophenol (0.20 mL, 1.95 mmol) and catalyst (1 mol %) were dissolved in CH_2_Cl_2_ (1.7 mL). Cyclohex-2-enone (0.15 g, 1.52 mmol) was added in one portion, and the resulting reaction mixture was stirred at room temperature for 2.5 h. The crude reaction mixture was diluted with CH_2_Cl_2_ and filtered, and the polymer beads were washed with CH_2_Cl_2_ (70 mL). The combined organic phase was evaporated in vacuo, and the crude product was purified by flash chromatography on silica gel (5% EtOAc in hexanes) to give the product as a colorless oil. This is a known compound [17]. Enantiomeric excess was determined by HPLC analysis (Chiralpak AD-H, 2% iPrOH in isohexane, 1.0 mL/min): *t*_R_ = 19.2 min and 26.2 min.

**General procedure for asymmetric Michael addition of methyl malonate to *trans*-β-nitrostyrene**: *trans*-β-Nitrostyrene (83.4 mg, 0.56 mmol) and methyl malonate (0.23 g, 1.78 mmol) were dissolved in toluene (1 mL). Catalyst **12** (10 mol %) was added, and the reaction mixture was left at −30 °C for 72 h. The crude reaction mixture was diluted with CH_2_Cl_2_ and filtered, and the polymer beads were washed with CH_2_Cl_2_ (50 mL). The combined organic phase was evaporated in vacuo, and the crude product was purified by flash chromatography on silica gel (Gradient: 5–40% EtOAc in hexanes). This is a known compound [21]. The conversion of starting material was determined by ^1^H NMR analysis of the crude product. Enantiomeric excess was determined by HPLC analysis (Chiralpak AD-H, 30% iPrOH in isohexane, 1.0 mL/min): *t*_R_ = 7.3 min and 9.3 min.
